# Whole exome sequencing of a patient with suspected mitochondrial myopathy reveals novel compound heterozygous variants in *RYR1*

**DOI:** 10.1002/mgg3.280

**Published:** 2017-03-30

**Authors:** Patrick R. Blackburn, Duygu Selcen, Jennifer M. Gass, Jessica L. Jackson, Sarah Macklin, Margot A. Cousin, Nicole J. Boczek, Eric W. Klee, Elliot L. Dimberg, Kathleen D. Kennelly, Paldeep S. Atwal

**Affiliations:** ^1^ Center for Individualized MedicineMayo ClinicJacksonvilleFlorida; ^2^ Department of Health Sciences ResearchMayo ClinicJacksonvilleFlorida; ^3^ Department of NeurologyMayo ClinicRochesterMinnesota; ^4^ Department of Clinical GenomicsMayo ClinicJacksonvilleFlorida; ^5^ Center for Individualized MedicineMayo ClinicRochesterMinnesota; ^6^ Department of Health Sciences ResearchMayo ClinicRochesterMinnesota; ^7^ Department of Clinical GenomicsMayo ClinicRochesterMinnesota; ^8^ Department of Laboratory Medicine and PathologyMayo ClinicRochesterMinnesota; ^9^ Department of NeurologyMayo ClinicJacksonvilleFlorida

**Keywords:** CFTD, congenital fiber‐type disproportion, congenital myopathy, malignant hyperthermia, ryanodine receptor 1, *RYR1*

## Abstract

**Background:**

Pathogenic variants in ryanodine receptor 1 (*RYR1,* MIM# 180901) are the cause of congenital myopathy with fiber‐type disproportion, malignant hyperthermia susceptibility type 1, central core disease of muscle, multiminicore disease and other congenital myopathies.

**Methods:**

We present a patient with global developmental delay, hypotonia, myopathy, joint hypermobility, and multiple other systemic complaints that were noted early in life. Later she was found to have multiple bone deformities involving her spine, with severe scoliosis that was corrected surgically. She was also diagnosed with ophthalmoplegia, chronic hypercapnic respiratory failure, and hypertension. At 22 years of age she presented to the genetics clinic with a diagnosis of mitochondrial myopathy and underwent whole exome sequencing (WES).

**Results:**

Whole exome sequencing revealed two novel compound heterozygous variants in *RYR1* (c.7060_7062del, p.Val2354del and c.4485_4500del, p.Tyr1495X).

**Conclusion:**

Review of her clinical, pathologic, and genetic findings pointed to a diagnosis of a congenital myopathy with fiber‐type disproportion.

## Introduction

The congenital myopathies and muscular dystrophies are clinically and genetically heterogeneous disorders (Bönnemann et al. 2014). Because of the overlap in disease phenotype across many of these disorders, marked variability in clinical presentation, and varying modes of inheritance, determining the genetic diagnosis has become increasingly challenging, even with the advent of next generation sequencing in clinical practice (Bönnemann et al. 2014). Pathogenic variants in the ryanodine receptor 1 (*RYR1*, MIM# 180901) gene have been implicated in a number of different disorders including congenital myopathy with fiber‐type disproportion (CFTD, MIM# 255310).

The *RYR1* gene contains 106 exons that encode a homotetrameric calcium channel that controls communication between transverse‐tubules and the sarcoplasmic reticulum in skeletal muscle by regulating cytosolic Ca^2+^ levels and excitation–contraction coupling (Jungbluth 2007; Hernández‐Ochoa et al. 2015). RYR1 is required for normal development of muscle fibers, skin, and heart during embryogenesis and pathogenic variation is thought to result in altered properties of RYR1 and changes in calcium homeostasis that can lead to a number of pathological states. *RYR1* variants associated with susceptibility to malignant hyperthermia and central core disease are primarily dominant missense variants, and very few small deletions or duplications have been described (Klein et al. 2012). These variants produce hypersensitive channels (prone to activation by muscle fiber depolarization) as in malignant hyperthermia or leaky (Ca^2+^ dysregulation and depletion of Ca^2+^ from the sarcoplasmic reticulum) RYR1 channels as in classic central core disease (Hernández‐Ochoa et al. 2015). Autosomal recessive *RYR1*‐related myopathies on the other hand, often result from a compound heterozygous missense variant in combination with a nonsense, splice‐site, or frameshift variant (Klein et al. 2012). These variants can cause excitation‐contraction uncoupling in the severe recessive from of central core disease or loss of normal *RYR1* expression as in multiminicore disease (Hernández‐Ochoa et al. 2015). As whole exome and whole genome sequencing are increasingly utilized, *RYR1* variants are being identified more frequently and underlie a significant proportion of neuromuscular disease cases.

Pathogenic variants in *RYR1*,* TPM3* (MIM# 191030), *TPM2* (MIM# 190990)*, ACTA1* (MIM# 102610), *SEPN1* (MIM# 606210)*, LMNA* (MIM# 150330)*, and MYH7* (MIM# 160760) genes can cause CFTD, with *TPM3* being the most common cause of this form of myopathy (Clarke et al. 2010; North et al. 2014). Clarke et al. (2010) described four families with the same pattern of recessive *RYR1* variants including one frameshift or truncating variant together with a missense change who had CFTD. The pathology and clinical phenotypes of patients with CFTD can be present in core myopathy, and it can be difficult to distinguish between these disorders (Clarke et al. 2010). It is also known that cores become more prominent with age, and rebiopsy of patients later in life can lead to different pathological findings (Clarke et al. 2010).

In this report, we describe a patient who was diagnosed with a mitochondrial myopathy early in life. After a protracted diagnostic odyssey, whole exome sequencing (WES) revealed novel compound heterozygous frameshift and in‐frame deletion variants in *RYR1* and further pathological characterization revealed this to be CFTD. While the patient described in this report has a phenotype consistent with autosomal recessive *RYR1*‐related congenital myopathy, it highlights the importance of this condition in the differential diagnosis for mitochondrial myopathy.

### Clinical description

The patient is a 22‐year‐old African American female with a complicated past medical history since birth involving myopathy, weakness, joint hypermobility, and multiple other systemic complaints that were attributed initially to a mitochondrial myopathy. The patient was born to term after an uncomplicated pregnancy with no known exposures. The patient had a normal birth weight of 6 lbs 11 oz and her Apgar scores were 7 at 1 min and 8 at 5 min. She was unresponsive after birth and was given oxygen via hood for 10 days. After 3 days, she was transferred to the neonatal intensive care unit (NICU) and was diagnosed with hypotonia and cerebral palsy. She had failure to thrive with trouble sucking and swallowing and at 10 months of age had a J‐tube placed. She remained tube‐dependent until she was 3 years old. She received physical, occupational, and speech therapy for global developmental delay. The patient never crawled, and she walked initially using a walker while working with a physical therapist at around 2 years of age. She also had speech delay and did not start speaking until she was 2 years old.

**Figure 1:**
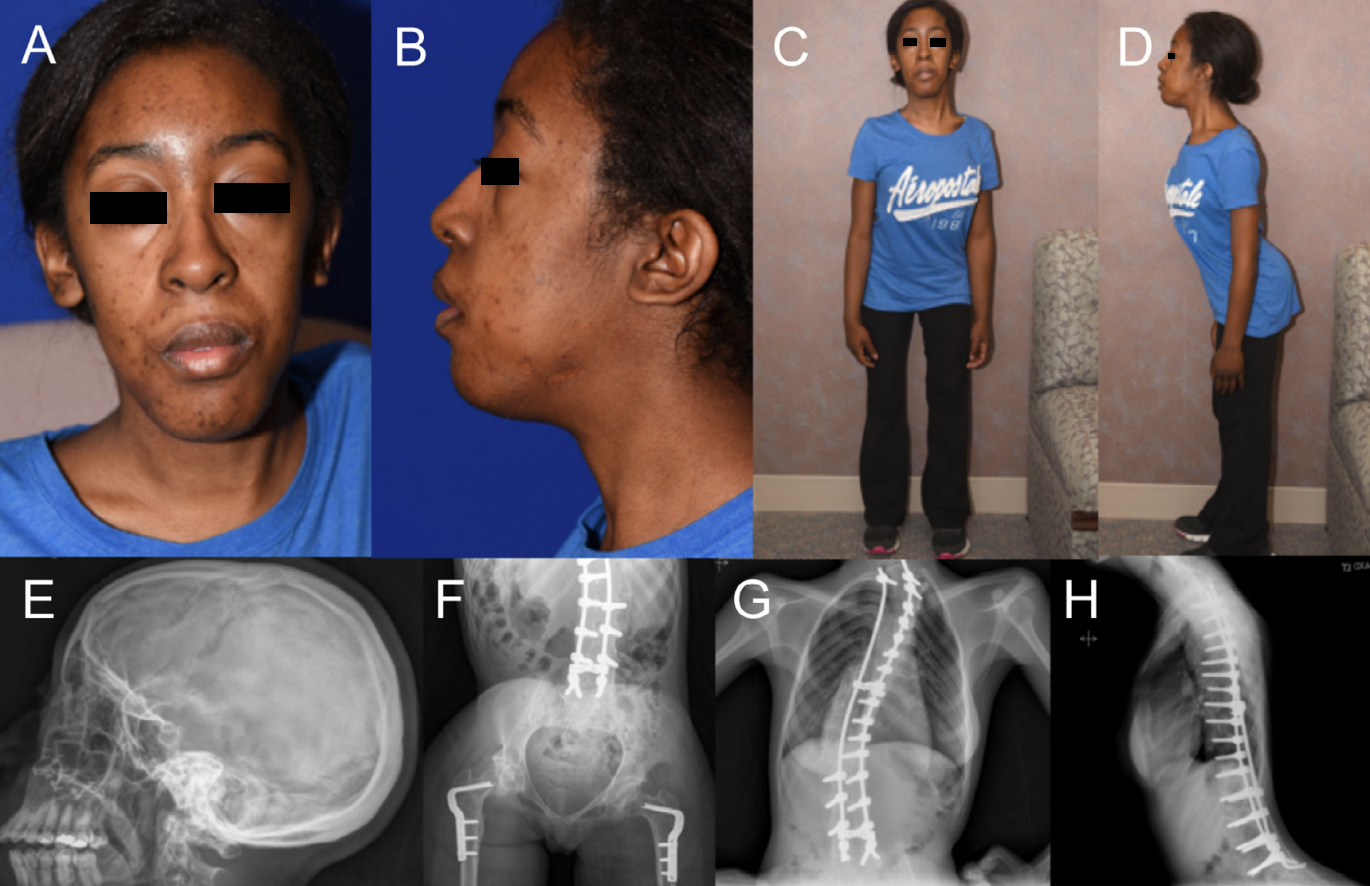
Patient photographs and skeletal survey. Photographs show mild ptosis and slight facial dysmorphism (A, B). Kyphoscoliosis is evident (C, D). Patient had maxillary surgery due to facial dysmorphism and teeth misalignment. Bilateral femoral osteotomy was performed. Pronounced scoliosis of the thoracolumbar spine with postsurgical changes of the posterior rods and bilateral pedicle screws traversing the thoracic and lumbar spine are evident. Skeletal abnormalities were secondary to congenital myopathy.

As a young child, she was diagnosed with external ophthalmoplegia with little upward eye movement, limited lateral gaze, and mild ptosis (Recent photographs are shown in Fig. 1A–D). She is reported to have had a muscle biopsy when she was 3 years old that showed evidence of oxidative phosphorylase deficiency and was given a diagnosis of a mitochondrial disorder. The patient was placed on CoQ10, carnitine, levocarnitine, and other supplements, which did not help her symptoms.

She also had multiple bone deformities involving her back, with mandibular abnormalities and severe scoliosis that required her to use a wheelchair. Secondary to her scoliosis and chest wall deformities she developed restrictive lung disease, obstructive and central apnea, frequent pneumonia, episodes of acute respiratory failure requiring mechanical ventilation, and hypertension. She also had episodes of tachycardia and postural orthostatic tachycardia syndrome (POTS) was suspected. She had maxillary surgery due to facial dysmorphism and teeth misalignment (Fig. 1E). She also had dysplastic changes involving the pelvis and acetabula for which she underwent a bilateral femoral osteotomy (Fig. 1F). The patient had rods and screw fixation in the upper thoracic and lower lumbar spine at age 11 for her scoliosis (Fig. 1G,H). Since high school, she has not been able to flex her neck and, due to weakness, must hold her head upright while she walks.

Recently, the patient became ill with the flu and was hospitalized for 3 days and has noticed a worsening of her condition since that time including extreme exhaustion, tachycardia, and increased lower extremity weakness. The patient started using a wheelchair again and was unable to walk as much as she used to due to weakness and fatigue. She was referred for evaluation of her neck and back pain, which was described as a chronic ache in her mid‐cervical spine that extends to her mid‐thoracic region. She has a lordotic, stiff gait but was able to stand on her toes and heels (Fig. 1C,D). She described progressive weakness of her lower extremities, a decrease in hip flexion, which together with the increasing weakness, had led to balance issues.

### Family history

**Figure 2:**
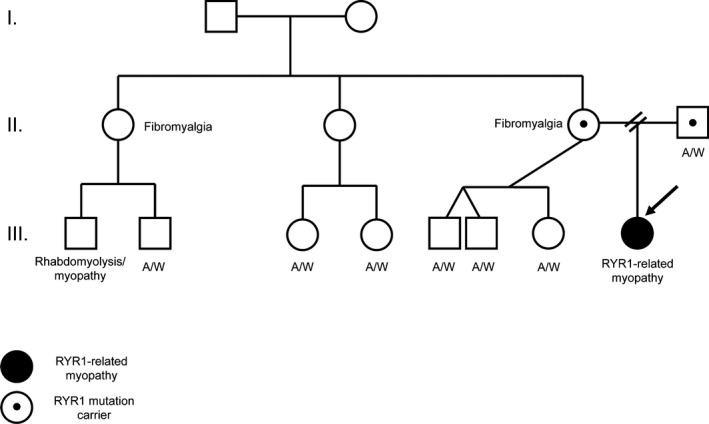
Family Pedigree. A three‐generation family pedigree showing the proband (arrow) and relatives. Note that each parent carries a different *RYR1* variant. Both the proband's mother and a maternal aunt have fibromyalgia and a maternal cousin has an undiagnosed myopathy with rhabdomyolysis.

A three‐generation family pedigree was obtained (Fig. 2). No family members are known to have similar phenotype. One first cousin has rhabdomyolysis and muscle disease; he is currently undergoing genetic testing. The paternal grandmother has an eye abnormality.

### Electromyography findings

Nerve conduction studies of the left upper and lower limbs revealed a borderline ulnar sensory response peak latency. Concentric needle examination of selected left upper and lower limb muscles demonstrated rapid recruitment of short duration, low amplitude, complex motor unit potentials, indicating a diffuse myopathy without abnormal spontaneous activity.

### Neuromuscular pathology

**Figure 3:**
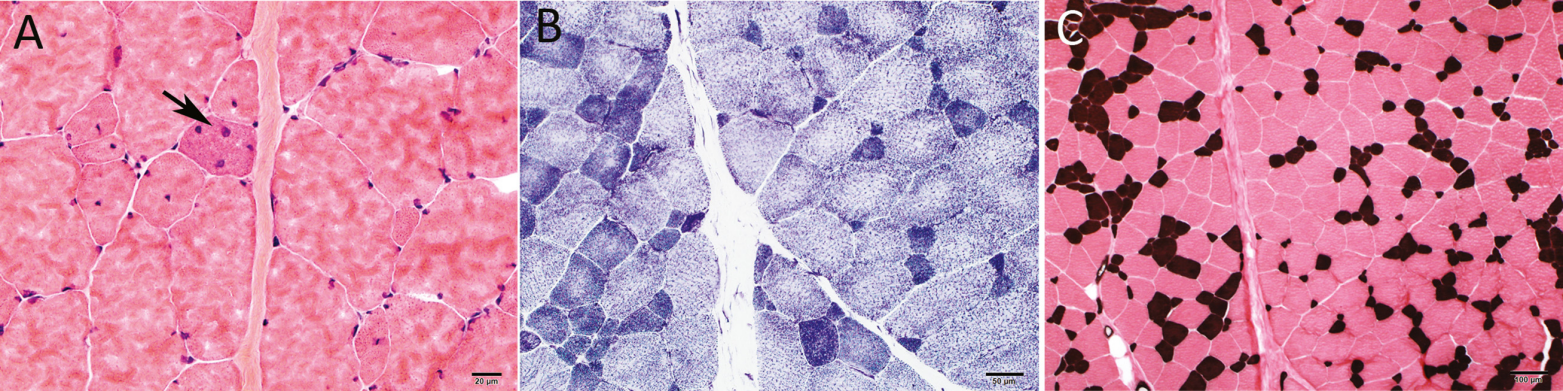
Histologic findings. Note a few fibers with internal nuclei and a regenerating fiber (arrow) (A); scattered fibers display irregularly circumscribed attenuations of oxidative enzyme activity (B); type 1 fibers have a smaller mean diameter than type 2 fibers and all atrophic fibers are type 1 (C). Section (A) is stained with hematoxylin and eosin, section (B) with NADH dehydrogenase, and section (C) is reacted for ATPase at pH = 4.3. Bars = 20 *μ*m in (A), 50 *μ*m in (B), and 100 *μ*m in (C).

A biopsy of the right vastus lateralis muscle was performed when the patient was 21 years of age. The muscle fibers varied pathologically from 5 to 100 micrometers in diameter. Fibers smaller than 25 micrometers occurred both singly and in small groups with up to five fibers per group. There was a mild increase of internal nuclei. Rare fibers were regenerating (Fig. 3A). No necrotic fibers were observed. There was a mild focal increase in perimysial fibrous connective tissue. In an NADH dehydrogenase reacted section, a few fibers displayed irregularly circumscribed decreases of enzyme activity (Fig. 3B). Type 1 fibers had a significantly smaller mean diameter than type 2 fibers, and all atrophic fibers were histochemically type 1 (Fig. 3C). Based on these findings, the patient was given a diagnosis of CFTD.

### Echocardiogram findings

An echocardiogram (ECG) was done to determine the etiology of tachycardia, hypertension, and suspected postural tachycardia (POTS). There was no evidence of POTS and left ventricular function was normal with a calculated ejection fraction of 69%. Her blood pressure is well controlled and the tachycardia is likely related to the severe deconditioning and restrictive pulmonary pattern seen in the patient.

### Laboratory findings

Laboratory studies including a mucopolysaccharide urine screen, urine organic acids, lactic acid, creatine kinase, acylcarnitine profile, and plasma amino acids were unrevealing.

## Methods

### Ethical compliance

The patient and her parents consented for sample collection and subsequent analysis under a protocol approved by the institutional review board of the Mayo Clinic. Consent was obtained from the patient to publish photographs.

### Whole exome sequencing methodology

Genomic DNA was extracted from blood from the proband, mother, and father. The Agilent Clinical Research Exome capture kit was used for exome enrichment and sequencing was done on an Illumina HiSeq 2000 that generates 100 bp paired‐end reads. Bi‐directional sequence was assembled, aligned to reference gene sequences based on human genome build GRCh37/UCSC hg19, and analyzed for sequence variants using a proprietary analysis tool (Xome Analyzer, GeneDx, Gaithersburg, MD, USA). Sanger sequencing was used to confirm all potentially pathogenic variants identified in this individual and in the parental samples. Sequence alterations were reported according to the Human Genome Variation Society (HGVS) nomenclature guidelines. The exome was covered to a mean depth of 91x, with a quality threshold of 95.6%.

The clinical analysis for the proband included evaluation of variants that were identified to be de novo, compound heterozygous, homozygous, heterozygous and X‐linked and prioritization of variants was based on the family structure and reported phenotype. Analysis in this case specifically included review of variants in genes associated with myopathy, mitochondrial disease, oxidative phophorylation deficiency, skeletal dysplasia, scoliosis, anemia, asthma, dysphagia, dyspnea, exercise intolerance, fatigue, facial dysmorphism, failure to thrive in infancy, hypotonia, global developmental delay, growth delay, headache, hypertension, joint hypermobility, malnutrition, muscle weakness, obstructive sleep apnea, central apnea, oligohydramnios, ophthalmoplegia, ptosis, orthostatic tachycardia, and tachycardia.

## Results

Whole exome sequencing (XomeDx, GeneDx) revealed a likely pathogenic 3 bp deletion (Chr19(GRCh37): g.38990307_38990309del, NM_000540.2: c.7060_7062del, NP_000531.2: p.Val2354del) in exon 44 of *RYR1* resulting in the in frame deletion of a highly conserved valine residue within the intracellular calcium release channel domain and was inherited from the patient's mother (Table**** 1). A second pathogenic deletion (Chr19(GRCh37): g.38969105_38969120del, NM_000540.2: c.4485_4500del, NP_000531.2: p.Tyr1495X) in *RYR1* leads to a premature stop in exon 31 (SPIa/RYanodine Receptor SPRY domain) and was detected in the patient's father (Table**** 1). A patient with a c.4485G>A missense variant that results in an identical premature stop codon (p.Tyr1495X) in *RYR1* has been previously described in Klein et al. in an individual who presented >10 years of age with proximal weakness, scoliosis, and muscle biopsy that revealed type 1 predominance in both cores and minicores (Klein et al. 2012). This individual also had two other missense variants of uncertain significance (VUS) in *RYR1* including c.1453A>G; p.Met485Val and c.325C>T; p.Arg109Trp. Neither of our patient's variants have been seen in approximately 6500 individuals of European and African American ancestry in the NHLBI Exome Sequencing Project nor in the 60,706 unrelated individuals in the ExAC database (Table**** 1) (Project NGES, 2012; Lek et al. 2016).

**Table 1:** Whole exome sequence and comprehensive mitochondrial nuclear gene panel results. Table showing the location of variants found in the proband including cDNA change, protein change, in silico prediction algorithm results, zygosity, mode of inheritance, association with disease, ACMG variant classification, population frequency (ExAC and ESP), rs number, and ClinVar accession number

Test	Gene	RefSeq Accession Number	cDNA change	Protein Change	SIFT	PolyPhen‐2	MutationTaster2	Zygosity	Inheritance	Mode of Inheritance	OMIM	ACMG classification	ExAC Frequency	ESP Frequency	DbSNP	ClinVar Accession
GeneDx XomeDx/Whole Exome Sequence Analysis	RYR1	NM_000540.2	c.7060_7062del	p.Val2354del	N/A	N/A	N/A	Heterozygous	Maternal	AD/AR	Central core disease MIM: 117000; Neuromuscular disease, congenital, with uniform type 1 fiber MIM: 117000; King‐Denborough syndrome MIM: 145600; Minicore myopathy with external ophthalmoplegia MIM: 255320; {Malignant hyperthermia susceptibility 1} MIM: 145600	Likely Pathogenic Variant	N/R	N/R		N/R
GeneDx XomeDx/Whole Exome Sequence Analysis	RYR1	NM_000540.2	c.4485_4500del	p.Tyr1495X	N/A	N/A	N/A	Heterozygous	Paternal	AD/AR	Central core disease MIM: 117000; Neuromuscular disease, congenital, with uniform type 1 fiber MIM: 117000; King‐Denborough syndrome MIM: 145600; Minicore myopathy with external ophthalmoplegia MIM: 255320; {Malignant hyperthermia susceptibility 1} MIM: 145600	Pathogenic Variant	N/R	N/R		N/R
GeneDx Comprehensive Mitochondrial Nuclear Gene Panel	MT‐CO2	NC_012920.1	c.136C>T	p.Leu46Phe	Tolerated	Probably Damaging	N/A	Homoplasmic	N/A	MT	Cytochrome c oxidase deficiency MIM: 220110 N/R	VUS	N/A	N/A		N/R
GeneDx Comprehensive Mitochondrial Nuclear Gene Panel	SARS2	NM_001145901.1	c.14T>C	p.Met5Thr	Tolerated	Benign	Polymorphism	Heterozygous	Paternal	AR	Hyperuricemia, pulmonary hypertension, renal failure, and alkalosis, HUPRA Syndrome MIM: 613845	VUS	0.00000008	N/R	rs538446780	N/R
GeneDx Comprehensive Mitochondrial Nuclear Gene Panel	AGK	NM_018238.3	c.416C>G	p.Thr139Arg	Tolerated	Benign	Polymorphism	Heterozygous	Maternal	AR	Autosomal recessive cataract 38 MIM: 614691; Sengers syndrome MIM: 212350	VUS	0.00000448	EA: G = 0.00% – AA: G = 0.43%	rs144706178	N/R
GeneDx Comprehensive Mitochondrial Nuclear Gene Panel	TIMM44	NM_006351.3	c.1340G>C	p.Ser447Thr	Tolerated	Benign	Disease causing	Heterozygous	N/A	N/R	N/R	VUS	0.00001612	EA: G = 0.00% – AA: G = 1.79%	rs7257461	N/R
GeneDx Comprehensive Mitochondrial Nuclear Gene Panel	PNPT1	NM_033109.4	N/A	N/A	N/A	N/A	N/A	Het	N/A	AR	Combined oxidative phosphorylation deficiency 13 MIM: 614932; autosomal recessive deafness 70 MIM: 614934	VUS	N/R	N/R		N/R

N/A, not available; N/R, not reported; VUS, variant of uncertain significance.

Previous sequence and deletion/duplication analysis of the mitochondrial genome and 139 nuclear genes associated with mitochondrial disorders (GeneDx) for this individual identified several heterozygous VUSs including a p.Leu46Phe homoplasmic VUS in mitochondrially encoded cytochrome C oxidase subunit II (*MT‐CO2*, MIM# 516040) (NC_012920.1: m.7721C>T, NC_012920.1: c.136C>T, p.COX2: Leu46Phe), a p.Met5Thr heterozygous VUS in seryl‐tRNA ligase (*SARS2*, MIM# 612804) (Chr19(GRCh37): g.39421363A>G, NM_001145901.1: c.14T>C, NP_001139373.1: p.Met5Thr), a p.Thr139Arg heterozygous VUS in acylglycerol kinase (*AGK*, MIM# 610345) (Chr7(GRCh37): g.141313971C>G, NM_018238.3: c.416C>G, NP_060708.1: p.Thr139Arg), a p.Ser447Thr heterozygous likely benign variant in translocase of inner mitochondrial membrane 44 homolog (*TIMM44*, MIM# 605058) (Chr19(GRCh37): g.7992091C>G, NM_006351.3: c.1340G>C, NP_006342.2: p.Ser447Thr, and a duplication of exons 1‐4 in polyribonucleotide nucleotidyltransferase 1 (*PNPT1*, MIM# 610316) (Table**** 1).

Comparison of the mitochondrial and nuclear genome panel and the whole exome sequencing results showed the *SARS2* variant to be paternally inherited and the *AGK* variant to be maternally inherited. No other potentially pathogenic variants were observed in the *SARS2* gene at 100% coverage of the coding region of this gene or the *AGK* gene at 96.3% coverage. Due to the lack of phenotypic overlap with these disorders, the *RYR1* variants identified are likely responsible for the patient's phenotype (Table 1).

## Discussion

As next generation sequencing becomes increasingly utilized in the clinic, more and more cases of *RYR1*‐related congenital myopathy are being uncovered. *RYR1* congenital myopathies are highly clinically variable and have a broad phenotypic spectrum with overlap with numerous other neuromuscular disorders with diverse molecular etiologies. These disorders can have neonatal or early childhood onset and the clinical manifestations may include neonatal hypotonia, delayed motor development, muscle weakness with feeding difficulty and failure to thrive in some cases (Beggs and Agrawal 2013). Scoliosis and secondary complications including respiratory impairment can also occur (Beggs and Agrawal 2013). The spectrum of *RYR1*‐related myopathies is also expanding with the recent characterization of polyhydramnios and fetal akinesia leading to arthrogryposis multiplex congenita, also known as lethal multiple pterygium syndrome (Kariminejad et al. 2016). Whole exome sequencing and next generation sequencing panels are extremely useful in unraveling the cause of complex neuromuscular disease and identifying the variants involved in a more efficient and cost‐effective manner as demonstrated in this case.

Through the integration of the clinical findings including muscle biopsy, genetic results, and other assessments, we were able to determine the correct underlying gene defect and pathological diagnosis of CFTD in this individual after a lengthy diagnostic odyssey. The two variants seen in this individual (p.Val2354del and p.Tyr1495X) have not been reported previously, but given her clinical presentation and pathologic diagnosis, they are likely to be pathogenic. Pathogenic variants in *RYR1* are typically associated with core myopathies (Amburgey et al. 2013). However recent evidence suggests that up to 50% of recessive *RYR1*‐related myopathies may exhibit non‐core pathology (Amburgey et al. 2013). Multiminicore disease, centronuclear myopathy, and CFTD are inherited in a recessive manner (Amburgey et al. 2013). Most patients with *RYR1*‐related myopathies present in infancy or early childhood and loss‐of‐function variants that result in reductions of RYR1 protein levels correlate with a more severe clinical presentation in patients (Dowling et al. 2012; Amburgey et al. 2013). Non‐central core pathology also seems to correlate with reduced RYR1 protein expression (Amburgey et al. 2013).

The p.Tyr1495X truncating variant uncovered in our patient likely undergoes nonsense mediated decay or results in a severely truncated non‐functional protein. The presence of CFTD (a non‐core pathology) found in this patient is in line with these previously observed associations. The p.Val2354del (3 bp in‐frame deletion) observed in our patient likely results in reduced RYR1 function, but additional studies will need to be done to confirm this. Several missense variants have been observed in neighboring residues adjacent to p.Val2354 in individuals with malignant hyperthermia and a 3 bp in‐frame deletion of a single residue (p.Glu2347) has been observed in two unrelated malignant hyperthermia‐susceptible families (Sambuughin et al. 2001). The p.Glu2347 in‐frame deletion in these families was evaluated using *in vitro* skeletal‐muscle‐contracture testing and found to produce a large electrically evoked contraction tension, suggesting that this small deletion leads to a significant alteration in skeletal‐muscle calcium regulation (Sambuughin et al. 2001). Based on the proximity of the patient's variant to other previously reported variants associated with malignant hyperthermia, we recommended that precautions be taken for the patient and her mother to avoid this anesthetic‐drug‐induced hypermetabolic syndrome (Sambuughin et al. 2001).

Despite *RYR1*‐related congenital myopathy being slowly progressive or static in most cases, some patients may experience decreased respiratory function or even respiratory failure, significant cardiac impairment, and increasing axial and proximal muscle weakness, as was the case in the patient described in this report. Currently only supportive care for respiratory symptoms, physical therapy for muscle weakness and contractures, and orthopedic correction are used to manage the disorder, but new treatment options may be on the horizon. The patient described in this report was recently enrolled in a phase I and phase II efficacy study of N‐acetylcysteine for the treatment of *RYR1*‐congenital myopathy at the National Institutes of Health in Bethesda, MD.

## Conflicts of Interest

The authors have no conflicts of interest.
